# Editorial: TRAF Proteins in Health and Disease

**DOI:** 10.3389/fimmu.2019.00326

**Published:** 2019-02-26

**Authors:** Gail A. Bishop, Ali A. Abdul-Sater, Tania H. Watts

**Affiliations:** ^1^Department of Microbiology and Immunology, University of Iowa, Iowa City, IA, United States; ^2^Department of Internal Medicine, University of Iowa, Iowa City, IA, United States; ^3^Iowa City VA Health Care System, Iowa City, IA, United States; ^4^School of Kinesiology and Health Science, Faculty of Health, York University, Toronto, ON, Canada; ^5^Department of Immunology, University of Toronto, Toronto, ON, Canada

**Keywords:** TNFR signaling, TLR signaling, NLR signaling, cytokine signaling, antigen receptor signaling, NF-kB, inflammation, cancer

TRAF proteins are a family of signaling adaptors that play diverse roles in signaling by a broad range of receptors involved in immunity and inflammation. First identified as signaling adaptors downstream of TNFR2/CD120b (1), TRAFs have been implicated in regulating signaling by antigen receptors, cytokine receptors, and members of the TNFR superfamily as well as receptors of the innate immune system (2). New insights into the structure of the family as well as a wealth of new studies on regulation of signaling by TRAFs prompted the creation of this special topic. This collection of 11 papers highlights the role of TRAF proteins in diverse signaling pathways as well as their role in a number of biological and disease processes.

There are 7 mammalian TRAFs. TRAFs 1 through 6 share the conserved TRAF domain, responsible for hetero- and homo-oligomerization of TRAF proteins, as well as recruitment to TRAF motifs in the cytoplasmic domains of cell surface receptors, and certain cytoplasmic and nuclear proteins (Figure 1). Crystal structures are now available for all 6 TRAF proteins including several recently elucidated complexes of TRAFs with binding partners. As reviewed by Park, comparison of these structures has revealed conserved as well as unique features of binding among the different TRAF proteins. TRAF1 is unusual among TRAF proteins in lacking the RING domain shared by TRAFs 2–6 and the non-conventional TRAF7. Here, Edilova et al. review the role of TRAF1 as a positive regulator of signaling downstream of TNFRs such as CD40, 4-1BB, and LMP1, and as a negative regulator of TLR signaling. They also discuss the potential roles of TRAF1 in human diseases, including arthritis and cancer (Edilova et al.).

**Figure 1:**
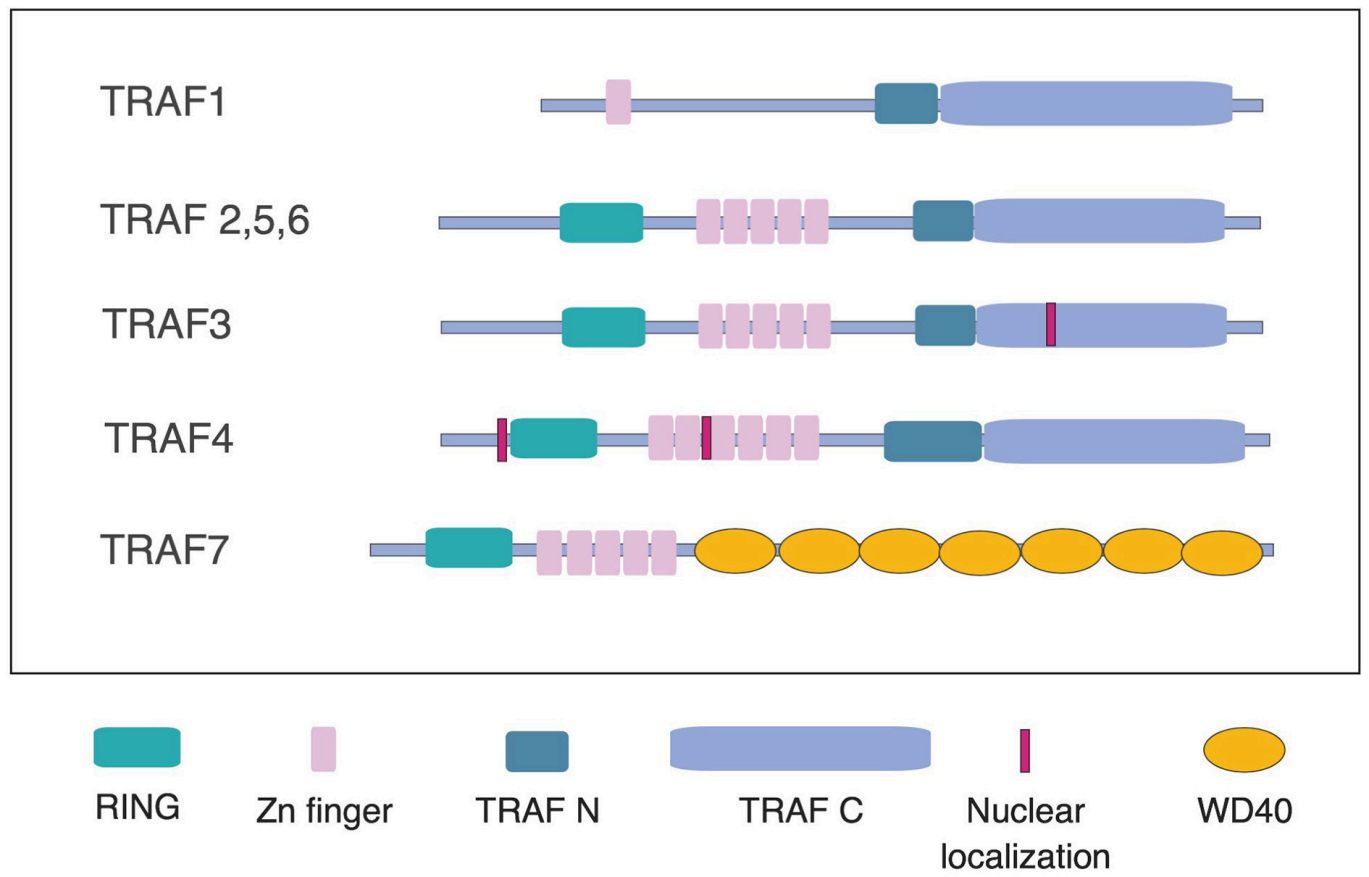
Schematic representation of TRAFs 1 through 7. TRAFs 2,3,5, and 6, share the conserved TRAF domain, consisting of the coiled-coil, TRAFN domain and the TRAF-C domain, also known as a MATH domain, as well as a RING domain and a series of Zn fingers. TRAF1 differs from the other TRAFs in lacking the RING domain. TRAF3 and TRAF4 have nuclear localization sequences. TRAF7 lacks the TRAF domain, which is replaced by a series of WD40 domains (adapted from reference Park).

NF-κB pathways—including both the canonical and non-canonical/NF-κB2 pathways—play important roles in the functions of TRAF proteins. These pathways, and related MAPK pathways, are the focus of a review by Shi and Sun. Shi and Sun discuss the role of TRAFs 2 and 6 as positive regulators of NF-κB and MAPK signaling, downstream of multiple receptors, the anti-inflammatory role of TRAF2 and 3 in restraining non-canonical NF-κB signaling, as well as TRAF3 as a negative regulator of TLR signaling.

TRAF proteins have important roles in regulation of inflammation through their role in activation of pattern recognition receptors, including Toll-like receptors, RIG-I like receptors, Nod-like receptors, inflammasomes, and STING signaling. In their mini-review, Dhillon et al. discuss the role of TRAFs in both positive and negative regulation of these pathways.

The physiologic importance of TRAFs 2, 3, and 6 was not easy to determine initially, because germline deletion of their genes in mice led to peri-natal lethality with multiple severe developmental abnormalities (3–6). The development of conditional deletion approaches has allowed much more information to be revealed about the *in vivo* functions of these molecules. This has in turn highlighted the cell-type-specific functions that characterize several of the TRAFs. Boyce et al. discuss the roles played by TRAF3 in regulating the balance between osteoblasts and osteoclasts in bone health, as well as disorders detrimentally impacting this balance. Their article summarizes studies showing that TRAF3 can both limit osteoclast formation by limiting signaling through the TNFR superfamily member RANK, as well as inhibiting TNF-induced osteoclast formation. The review by Bishop et al. demonstrates that TRAF3 regulates normal and malignant B cell biology via multiple mechanisms, including the pathways just mentioned as well as by modulating glucose metabolism, and inhibiting targets of the nuclear CREB complex.

Given the critical role of NF-κB in regulating genes associated with inflammation and cellular survival, it is not surprising that TRAF proteins play important roles in cancer. TRAF-dependent signaling pathways are altered in many cancers and there is extensive evidence for both genetic and post-translational alterations in TRAF signaling. In their review, Zhu et al. provide a comprehensive analysis of the Cancer Genome Atlas and the Catalog of Somatic Mutations in Cancer, with respect to genetic alterations in TRAF proteins in cancer. They find that all 7 TRAF family members show alterations in human cancers, with gain of function common for TRAFs 1, 4, 5, and 6, and loss of function commonly seen for TRAFs 3 and 5. In a related original research contribution, Perez-Chacon et al. describe a mouse model in which global overexpression of TRAF3 and Bcl2 results in tumors with features of mature Non-Hodgkin B cell lymphoma. The results suggest that TRAF3 and Bcl2 cooperate to induce neoplasms of mature B cells in mice. Although seemingly at odds with the finding that TRAF3 is frequently lost in human cancer, the review by Dhillon et al. points out that TRAF3 not only inhibits some aspects of TLR function, but can enhance inflammation downstream of TRIF dependent receptors, so TRAF3 can both positively and negatively regulate tumorigenesis. Additionally, it is now clear that TRAFs, particularly TRAF3, can play varied and even divergent cell-type specific functions. As the overexpression of TRAF3 in the mouse model studied in Perez-Chacon et al. is not confined to a single cell type, the phenotype of these mice likely reflects complex interactions between multiple TRAF3-overexpressing cell types.

T cell differentiation, leading to the development of a T follicular helper (Tfh) response, is critical for the development of germinal centers and affinity maturation of the B cell response (7). TRAFs are involved in signaling by a number of receptors that have been implicated in the development of the Tfh response, including the TNFR family members OX40, GITR, and 4-1BB, reviewed in this collection by Pedros et al. ICOS, a critical receptor for Tfh development, shares with TRAF proteins a TBK1 binding motif, and the authors discuss how this TRAF-mimicking signal plays a key role in Tfh development (Pedros et al.).

A more in depth look at the 4-1BB signalosome is provided by Zapata et al., who propose that different TRAF trimer configurations can allow formation of a complex, higher order signalosome with opportunities for recruitment of diverse signaling molecules. The authors also discuss the use of agonists against 4-1BB in cancer therapy and the implications of understanding 4-1BB signaling for design of new cancer treatments (Zapata et al.).

Signaling through the IL-6 receptor (IL-6R) plays an essential role in differentiation of CD4 T cells into Th17 cells (8), cells with important protective functions against extracellular bacteria and fungi (9). However, Th17 cells can also have a pathological role in diseases such as experimental autoimmune encephalomyelitis (EAE) (10), an experimental model for human multiple sclerosis. The IL-6R consists of two chains, the IL-6Rα chain and gp130, shared with other members of the IL-6R family. In this collection, Nagashima et al. discuss how IL-6R in naïve CD4 T cells binds TRAF2 and 5, thereby restricting the binding of JAKs to gp130, and limiting subsequent IL-6-mediated Stat3 activation. Thus, knockdown of TRAF2 or 5 enhances Th17 development and exacerbates EAE.

Overall, the articles in this Frontiers topic address the pivotal roles that TRAF proteins play as positive and negative regulators of inflammation and immunity mediated by a diverse array of receptors and cell types. The fact that each of the TRAF proteins can play both positive and negative roles in particular pathways or contexts, defies simple generalization. The dysregulation of TRAF proteins in cancer and a number of inflammatory diseases, suggests that this is an area that needs further attention. However, the nuanced role of TRAF proteins in each context will need to be carefully evaluated before they can be manipulated therapeutically.

## Author Contributions

All authors listed have made a substantial, direct and intellectual contribution to the work, and approved it for publication.

### Conflict of Interest Statement

The authors declare that the research was conducted in the absence of any commercial or financial relationships that could be construed as a potential conflict of interest.
